# Very-Long-Chain Acyl-Co-Enzyme A Dehydrogenase Deficiency Presenting as Rhabdomyolysis: First Case Report from Sri Lanka

**DOI:** 10.1155/2020/8894518

**Published:** 2020-10-13

**Authors:** Maheshi Wijayabandara, Champika Gamakaranage, Dineshani Hettiarachchi

**Affiliations:** ^1^University Medical Unit, National Hospital of Sri Lanka, Colombo, Sri Lanka; ^2^Department of Clinical Medicine, Faculty of Medicine, University of Colombo, Colombo, Sri Lanka; ^3^Human Genetics Unit, Faculty of Medicine, University of Colombo, Colombo, Sri Lanka

## Abstract

**Background:**

Rhabdomyolysis can be either inherited or acquired such as in metabolic myopathies. Very-long-chain acyl-CoA dehydrogenase deficiency is a rare fatty acid oxidation disorder which presents with different phenotypes, and the mild adult form can present as intermittent rhabdomyolysis. Here, we present the first adult case of very-long-chain acyl-CoA dehydrogenase deficiency presenting as rhabdomyolysis in a Sri Lankan patient. *Case Presentation*. A 36-year-old Sri Lankan man who was born to consanguineous parents presented with severe generalized muscle pain, stiffness, and dark-coloured urine for three days following prolonged low-intensity activity. Since fourteen years of age, he has had multiple similar episodes, where one episode was complicated with acute kidney injury. His eldest brother also suffered from the similar episode. Examination revealed only generalized muscle tenderness without any weakness. His creatine phosphokinase level was above 50,000 IU/L, and he had myoglobinuria. Molecular genetic tests confirmed the diagnosis of very-long-chain acyl-CoA dehydrogenase deficiency. Following a successful recovery devoid of complications, he remained asymptomatic with lifestyle adjustments.

**Conclusion:**

Very-long-chain acyl-CoA dehydrogenase deficiency is a rare inherited cause of metabolic myopathy that gives rise to intermittent rhabdomyolysis in adults. Prompt diagnosis is essential to prevent complications and prevent its recurrence.

## 1. Introduction

Rhabdomyolysis is a syndrome characterized by severe acute muscle injury causing muscle pain, weakness, dark-coloured urine, and the release of intracellular muscle constituents into the circulation [1]. Acquired causes such as vigorous exercise, trauma, alcohol, illicit substances, toxins, medications, autoimmune myopathies, electrolyte abnormalities, and endocrine disturbances are implicated in its aetiology. Some of the inherited causes are metabolic myopathies, channelopathies, and muscular dystrophies [1]. Metabolic myopathies such as glycogen storage disorders and disorders of fatty acid oxidation trigger rhabdomyolysis during exercise, fasting, and febrile illnesses [1]. Glycogen storage disorders are known to trigger rhabdomyolysis following short bursts of high-intensity exercise such as sprinting, with interictal weakness in adults and interictal elevated creatine phosphokinase (CPK) levels [2]. In contrast, disorders of fatty acid oxidation result in rhabdomyolysis after prolonged low-intensity exercise or fasting with interictal normal muscle power and CPK levels [2].

Carnitine palmitoyl transferase (CPT) II deficiency and very-long-chain acyl-CoA dehydrogenase (VLCAD) deficiency are recognized as causes of adult myopathic forms due to fatty acid metabolism disorders [2]. VLCAD is the key enzyme which catalyzes the first step of mitochondrial *β*-oxidation of long-chain fatty acids with a chain length of 14 to 20 carbons [3]. The human VLCAD gene has been identified and located on the short arm of chromosome 17 between p11.2 and p.11.13105 [4]. VLCAD deficiency is inherited as an autosomal recessive disorder presenting as three phenotypes [3]. These phenotypes differ according to the age of onset and severity. The early infantile onset form is severe with cardiac and multiorgan failure characterized by hypertrophic or dilated cardiomyopathy, pericardial effusion, hypotonia, and hepatomegaly [3]. The early childhood onset form is moderately severe presenting with hypoketotic hypoglycaemia and hepatomegaly without cardiac involvement [3]. The adult onset episodic myopathic form is less severe and presents with intermittent rhabdomyolysis triggered by prolonged exercise or fasting [3].

The incidence of VLCAD deficiency is estimated to be between 1 : 30,000 to 1 : 100,000 live births [3]. However, it is largely under-recognized and under-reported in Sri Lanka. Here, we report the first case of rhabdomyolysis in an adult male due to VLCAD deficiency in a patient of Sri Lankan origin.

## 2. Case presentation

**Figure 1:**
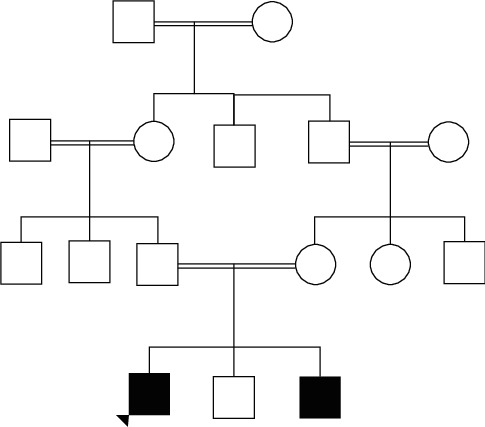
Pedigree chart of the patient. The pedigree chart shows the consanguineous marriages in three generations and the manifestation of the disease in our patient and possibly in his brother.

The proband 36-year-old Sri Lankan man was the third son born to a healthy first-degree consanguineous couple (Figure 1). He presented with severe generalized muscle pain, stiffness, and dark-coloured urine for three days. A day prior to his presentation, he had engaged in prolonged physical exertion (cleaning his apartment and lifting heavy weights). He has had previous episodes of intermittent generalized muscle pain and stiffness since the age of fourteen years, following sports activities at school and when he skipped meals. However, it had not come to medical attention. There were three similar episodes of generalized muscle pain, stiffness, and dark-coloured urine following prolonged low-intensity exercise at the ages of 20, 24, and 27 years. The second episode was treated at a local hospital which was complicated with acute kidney injury due to rhabdomyolysis without identifying the aetiology. The other two episodes lasted for about one week and resolved spontaneously.

His eldest brother (45 years) also suffered from a similar episode at the age of 20 years following a mountain hike. He experienced generalized muscle pain and dark-coloured urine. He was treated with haemodialysis and required ventilator support. However, aetiological diagnosis was not established. He subsequently had several milder episodes with muscle pain and stiffness related to physical exertion. His parents were healthy and had no symptoms suggestive of muscle disease.

The proband was not on any regular medications such as statin therapy and herbal medications and did not have any features suggestive of hypothyroidism. He did not have a recent history of trauma or infections. He occasionally consumed alcohol and smoked cigarettes at social events. However, he had no history of illicit substance abuse.

**Figure 2:**
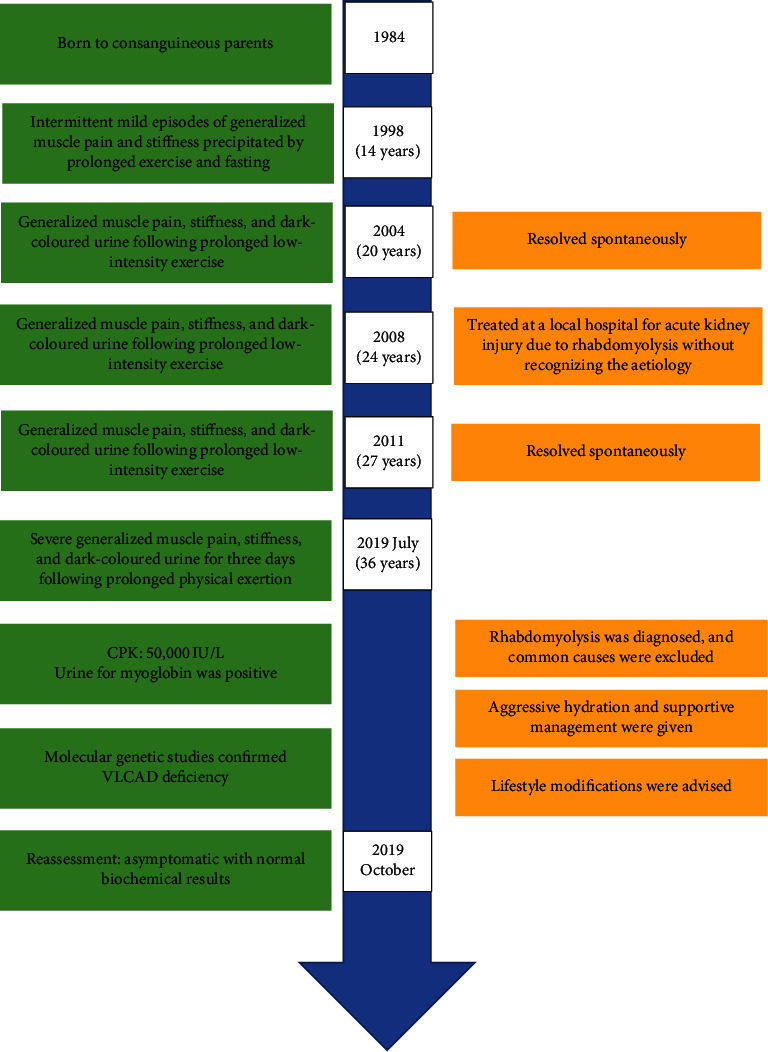
Timeline of events. Important events of the patient are shown in a timeline.

On examination, his body mass index was 24.1 kg/m^2^. He was afebrile and had generalized muscle tenderness. Neurological examination showed muscle power in upper limbs, lower limbs, and neck flexors as 5 (medical research council's scale) with normal reflexes, and vital capacity was 3.2 L. Cardiovascular, respiratory, and abdominal examinations were normal. The timeline of events are shown in Figure 2.

His biochemical and haematological investigations are shown in Table 1. On admission to the National Hospital of Sri Lanka, his serum creatinine was 1.8 mg/dL (reference range: 0.9–1.3 mg/dL), and urine for myoglobin was positive. Infectious disease screening including human immunodeficiency virus was negative. The clinical diagnosis of metabolic myopathy with rhabdomyolysis precipitated by exercise was suspected based on the clinical presentation, history of similar episodes, significant family history, and biochemical results. Furthermore, additional investigations revealed serum lactate of 1.32 mmol/L (reference range: 0.5–2.30) and serum ammonia of 33 *μ*mol/L (reference range: <40).

Molecular genetic studies were conducted using a customized gene panel consisting of the following genes: GYS1, GYS2, G6PC, SLC37A4, GAA, AGL, GBE1, PYGM, PYGL, PFKM, PHKA2, PGAM2, LDHA, ALDOA, ENO3, PHKB, PHKA1, PGM1, GYG1, PRKAG2, PHKG2, LPIN1, and ACADVL. The results revealed a homozygous pathogenic variant in the acyl-CoA dehydrogenase very-long-chain (ACADVL) gene: ACADVL, c.589G > A p. (Val197Met) confirming the genetic diagnosis of autosomal recessive VLCAD deficiency (OMIM : 201475). Additionally, a homozygous variant of uncertain significance was identified in the amylo-alpha-1,6-glucosidase, 4-alpha-glucanotransferase (AGL) gene: AGL, and c.3509A > G p. (Asn1170Ser), with a possible genetic diagnosis of autosomal recessive glycogen storage disease III a/b. Furthermore, no other clinically relevant variants were identified, and the copy number variant analysis was negative.

2D echocardiogram did not reveal hypertrophy or cardiomyopathy, and ultrasound scan of abdomen did not show hepatomegaly. He did not have any hypoglycaemic episodes.

He was aggressively hydrated with intravenous 0.9% NaCl while monitoring the urine output. Paracetamol was prescribed for pain relief. His biochemical parameters including serum creatinine improved. Since he neither progress to severe acute kidney injury nor develop electrolyte abnormalities, he was discharged after three days.

Our patient, a photographer by profession, did not have a regular eating pattern was accustomed to frequently skipping meals and engaged in hiking, playing cricket as recreational activities which precipitated most of the previous episodes. He was advised on lifestyle modifications to avoid prolong fasting, prolonged or strenuous exercise, dehydration, stress, and a high-fat diet [3]. He was specifically advised to have frequent low-fat, high-carbohydrate meals and was educated on the importance of early recognition of symptoms and prompt hospital admission to prevent acute kidney injury due to severe disease. Follow-up assessment done three months after discharge revealed that he was asymptomatic with a normal biochemical profile including a normal CPK level. Genetic screening for his two brothers who are currently living overseas was suggested. His parents, who were asymptomatic, did not undergo genetic screening due to financial restrains. He was advised on genetic counselling before marriage.

## 3. Discussion

We report a case of VLCAD deficiency presenting as rhabdomyolysis in an adult man, which is an underdiagnosed condition in Sri Lanka. The clinical presentation with generalized muscle pain, stiffness, and dark-coloured urine with high CPK levels suggested rhabdomyolysis. Common acquired causes of rhabdomyolysis such as infections, trauma, toxins, drugs, electrolyte, and endocrine disturbances were excluded. Onset of the symptoms of our patients was at the age of fourteen years which was followed by three episodes of clinically suggestive rhabdomyolysis of varying severity. Furthermore, as he was born to consanguineous parents and had a family history of a similar illness, a metabolic myopathy was considered as the aetiology for rhabdomyolysis. The current and previous episodes were triggered by prolonged low-intensity exercise, and he was asymptomatic in between the episodes, suggesting a disorder of fatty acid metabolism, the most likely aetiology.

CPT II deficiency is the most common disorder of fatty acid metabolism and a common metabolic cause for recurrent rhabdomyolysis [2]. VLCAD deficiency is rare but a recognized cause of metabolic myopathy leading to rhabdomyolysis [1]. As VLCAD is the key catalytic enzyme in the first step of mitochondrial *β*-oxidation of very-long-chain fatty acids, its deficiency leads to the inability of utilizing long-chain fatty acids, which is the main fuel source during prolonged exercise and fasting [3]. Consequently, the milder adult form of VLCAD deficiency presents as intermittent rhabdomyolysis during prolonged low-intensity exercise and fasting, probably due to some residual activity of the enzyme [1]. There are several cases reported in the literature describing the adult form of VLCAD deficiency presenting as rhabdomyolysis [5] and subsequent complications such as acute kidney injury [6] and respiratory failure [7].

VLCAD deficiency could be suspected biochemically by abnormal acylcarnitine levels such as accumulation of tetradecenoyl carnitine (C14 : 1), measured by tandem mass spectrometry during a stressful event [3]. Subsequently VLCAD deficiency could be confirmed by molecular genetic studies demonstrating biallelic pathogenic variants in ACADVL gene or by specialized biochemical tests such as analysis of fatty acid *β*-oxidation in cultured fibroblasts and by analyzing VLCAD enzymatic activity [3]. However, Sri Lanka, being a resource-limited country, acylcarnitine analysis or specialized biochemical tests is not performed currently. The molecular genetic analysis confirmed the diagnosis of VLCAD deficiency in our patient by identifying a homozygous pathogenic variant in the ACADVL gene comprising 20 exons spanning approximately 5.4 kb. There are more than 80 mutations in the ACADVL gene reported in the literature [8]. The genetic variant thus identified (c.3509A > G) in our patient resulted in a nonconservative amino acid change from valine to methionine at position 197 which has been described previously as pathogenic [9].

Furthermore, a homozygous variant of uncertain significance was identified in the AGL gene which was responsible for glycogen storage disease type III a/b. The clinical presentation of rhabdomyolysis following prolonged low-intensity exercise, asymptomatic “interictal” periods, and normal CPK levels at three months of assessment is more in favor of a VLCAD deficiency rather than a glycogen storage disorder.

The proband recovered completely without developing any complications. He adhered to lifestyle modifications and remained asymptomatic at three months. Due to the availability of limited resources, VLCAD deficiency is not diagnosed in Sri Lanka before. Unawareness and lack of access to genetic testing made it so delayed in diagnosis of this condition in our patient despite recurrent episodes. Therefore, we hope that this case will help clinicians to arrive at a prompt diagnosis when a patient presents with features of VLCAD deficiency as a rare cause for rhabdomyolysis and to advise on lifestyle adjustments to prevent further episodes.

Genetic testing and identification of VLCAD deficiency in family members is of utmost importance in preventing future complications, as severe form of rhabdomyolysis could be precipitated by prolonged activity, fasting, and even by anaesthetic agents that contain high doses of long-chain fatty acids such as propofol and etomidate [3]. Furthermore, such patients should be screened for hypothyroidism and should be advised against the use of statins and fibrates which could aggravate the existing muscle disease.

## 4. Conclusions

Inherited metabolic myopathy as an aetiology for rhabdomyolysis should be considered after exclusion of common causes and especially in those patients with a positive family history and consanguinity. VLCAD deficiency which is a rare disorder of fatty acid oxidation presents in adults as a milder form with episodic muscle pain and weakness with intermittent rhabdomyolysis following prolonged activity and fasting. Prompt diagnosis based on history, biochemical, and molecular genetic testing is crucial in the management and prevention of further recurrence and life-threatening complications.

## Figures and Tables

**Table 1:** Biochemical and haematological parameters of the patient.

Laboratory parameter	Value	Reference range
Biochemistry		
Creatine phosphokinase (U/L)	50,454	<195
Lactate dehydrogenase (U/L)	2209	230–460
Aspartate aminotransferase (U/L)	1557	<38
Alanine aminotransferase (U/L)	254	<40
Serum creatinine (mg/dL)	0.75	0.9–1.3
Serum potassium (mmol/L)	4.3	3.5–5.1
Serum sodium (mmol/L)	138	135–148
Serum calcium (mmol/L)	2.3	2.2–2.7
Serum phosphate (mmol/L)	1.3	0.8–1.5
C-reactive protein (mg/L)	5	<6
Serum thyroid stimulating hormone (mIU/L)	1.58	0.4–4
Fasting blood sugar (mg/dL)	94	100–125

Haematology		
Total white cell count (cells/*μ*L)	9690	4000–11,000
Neutrophil count (cells/*μ*L)	4400	1500–8,000
Lymphocyte count (cells/*μ*L)	3860	1000–4,800
Haemoglobin level (g/dL)	14.2	13.5–17.5
Platelet count (platelets/*μ*L)	242,000	150,000–450,000
Erythrocyte sedimentation rate (mm/1^st^ hour)	5	—

## Data Availability

The data used to support the findings of this study are included within the article.

## Abbreviations

CPK: Creatine phosphokinase
CPT: Carnitine palmitoyl transferase
VLCAD: Very-long-chain acyl-CoA dehydrogenase
ACADVL: Acyl-CoA dehydrogenase very-long-chain
AGL: Amylo-alpha-1,6-glucosidase, 4-alpha-glucanotransferase.
